# Ultrafast cold-brewing of coffee by picosecond-pulsed laser extraction

**DOI:** 10.1038/s41538-022-00134-6

**Published:** 2022-04-08

**Authors:** Anna R. Ziefuß, Tim Hupfeld, Sven W. Meckelmann, Martin Meyer, Oliver J. Schmitz, Wiebke Kaziur-Cegla, Lucie K. Tintrop, Torsten C. Schmidt, Bilal Gökce, Stephan Barcikowski

**Affiliations:** 1 Technical Chemistry I and Center for Nanointegration Duisburg-Essen (CENIDE), University of Duisburg-Essen, Universitaetsstrasse 7, 45141 Essen, Germany; 2 Applied Analytical Chemistry, University of Duisburg-Essen, Universitaetsstrasse 5, 45141 Essen, Germany; 3 Instrumental Analytical Chemistry, University of Duisburg-Essen, Universitaetsstrasse 5, 45141 Essen, Germany; 4 Centre for Water and Environmental Research, University of Duisburg-Essen, Universitaetsstrasse 5, 45141 Essen, Germany; 5 Materials Science and Additive Manufacturing, University of Wuppertal, Gaußsstr. 20, 42119 Wuppertal, Germany

**Keywords:** Science, technology and society, Synthesis and processing

## Abstract

Coffee is typically brewed by extracting roasted and milled beans with hot water, but alternative methods such as cold brewing became increasingly popular over the past years. Cold-brewed coffee is attributed to health benefits, fewer acids, and bitter substances. But the preparation of cold brew typically needs several hours or even days. To create a cold-brew coffee within a few minutes, we present an approach in which an ultrashort-pulsed laser system is applied at the brewing entity without heating the powder suspension in water, efficiently extracting caffeine and aromatic substances from the powder. Already 3 min irradiation at room temperature leads to a caffeine concentration of 25 mg caffeine per 100 ml, comparable to the concentrations achieved by traditional hot brewing methods but comes without heating the suspension. Furthermore, the liquid phase’s alkaloid content, analyzed by reversed-phase liquid chromatography coupled to high-resolution mass spectrometry, is dominated by caffeine and trigonelline and is comparable to traditional cold-brewed coffee rather than hot-brewed coffee. Furthermore, analyzing the head-space of the prepared coffee variants, using in-tube extraction dynamic head-space followed by gas chromatography coupled to mass spectrometry, gives evidence that the lack of heating leads to the preservation of more (semi-)volatile substances like pyridine, which provide cold-brew coffee its unique taste. This pioneering study may give the impetus to investigate further the possibility of cold-brewing coffee, accelerated by more than one order of magnitude, using ultrafast laser systems.

## Introduction

The worldwide coffee consumption is growing at an annual rate of 20% and amounting to 9.6 billion kg in 2018^1^. The taste of coffee is individual and is determined by many different factors. In addition to the type of the coffee bean and the roasting process, the subsequent handling is essential. Hot-brewed filter coffee is probably the most popular brewing method. However, since cold-brewed coffee is being successfully marketed at popular coffeehouse chains, this brewing method experienced growing enthusiasm in the past years, as indicated by an increase of 460% in retail sales from 2015–2017 in the United States^2^. In contrast to hot coffee, cold brew is prepared at or below room temperature and takes several hours to days for good extraction of caffeine and other aromatic substances^3,4^. In general, the chemical composition of coffee is highly dependent on the brewing method^5^. It is suspected that the cold-brewing method leads to less acidity and less bitterness than the hot brewing method^5–7^, making cold-brew coffee a lifestyle product with various recipes published in health blogs, magazines, and social media. As a limiting disadvantage, the time-consuming preparation of cold brew remains, which makes consumption less flexible and spontaneous compared to hot brew. In addition, product development is slowed down due to these long extraction times. Although several machines have been developed to automate the brewing process^8–10^, the overall process time could not be significantly reduced and is still about 12 h. Increasing caffeine and aroma release by increasing the available surface of powder by further grinding only works to a certain degree since typical filters do not work with too small particles. Furthermore, the clogging of the filters increases the processing time even more. To overcome these limitations, a completely different approach for combining cold-brew coffee’s lifestyle and health benefits with flexibility and fast preparation is necessary.

Our promising approach is based on the scalable method of laser synthesis and processing of colloids (LSPC)^11^, which is originally a method to maintain pure inorganic nanoparticles from metals^12^, semiconductors^13^, or oxides^14^. One way to perform LSPC is by laser post-processing (LPP) of particle suspensions, e.g., causing fragmentation induced by thermal or electronic processes which depend on laser parameters such as laser wavelength^15^, pulse duration^16^, repetition rate^17^, and pulse energy^18^. Furthermore, disaggregation and the break-up of porous particles by laser-induced shockwaves were reported^19^. However, the structure of organic solids differs from that of inorganic particles, and so far, there is only limited literature on the LSPC of the former^20,21^. A few reports can be found where a pulsed laser was used to dissolve or fragment bioactive substances, such as insoluble drugs^22,23^, curcumin^24^, or cinnamon^25^. An advantage of LPP in contrast to traditional milling processes is the contact-free fragmentation, which prevents contamination (e.g., by grinding media abrasion), is a tool that does not wear and leads to high reproducibility. Furthermore, it belongs to the class of digital photonic production processes that can easily be automated or remotely controlled^26^.

Within the mentioned studies, laser fragmentation leads to a decrease in particle size, accompanied by a higher bioavailability of the included organic lipophilic substances^27^. On the other side, organic material can be chemically broken and degraded by laser irradiation, as shown by Sylvestre et al. for fragmentation of megestrol acetate^20^. Such degradation of organic substances is almost neglectable (<2%), similar to the degradation that grinding processes cause. This minimized degradation is expected for femtosecond or picosecond pulses, where the short pulse duration prevents electron-phonon coupling and heat propagation into the substrate.

In this work, we transfer the method of LPP to the brewing of coffee to decrease the time to prepare fresh cold-brew coffee. Besides, we studied the head-space and liquid phase of hot-brewed, cold-brewed, and laser-generated coffee and compared them. The detailed chemical analysis reveals similar compositions of laser-extracted and conventionally cold-brewed coffee. Furthermore, the ultrafast laser method outperforms both hot and cold brewing in the caffeine release per minute by two orders of magnitude.

## Results and discussion

**Fig. 1:**
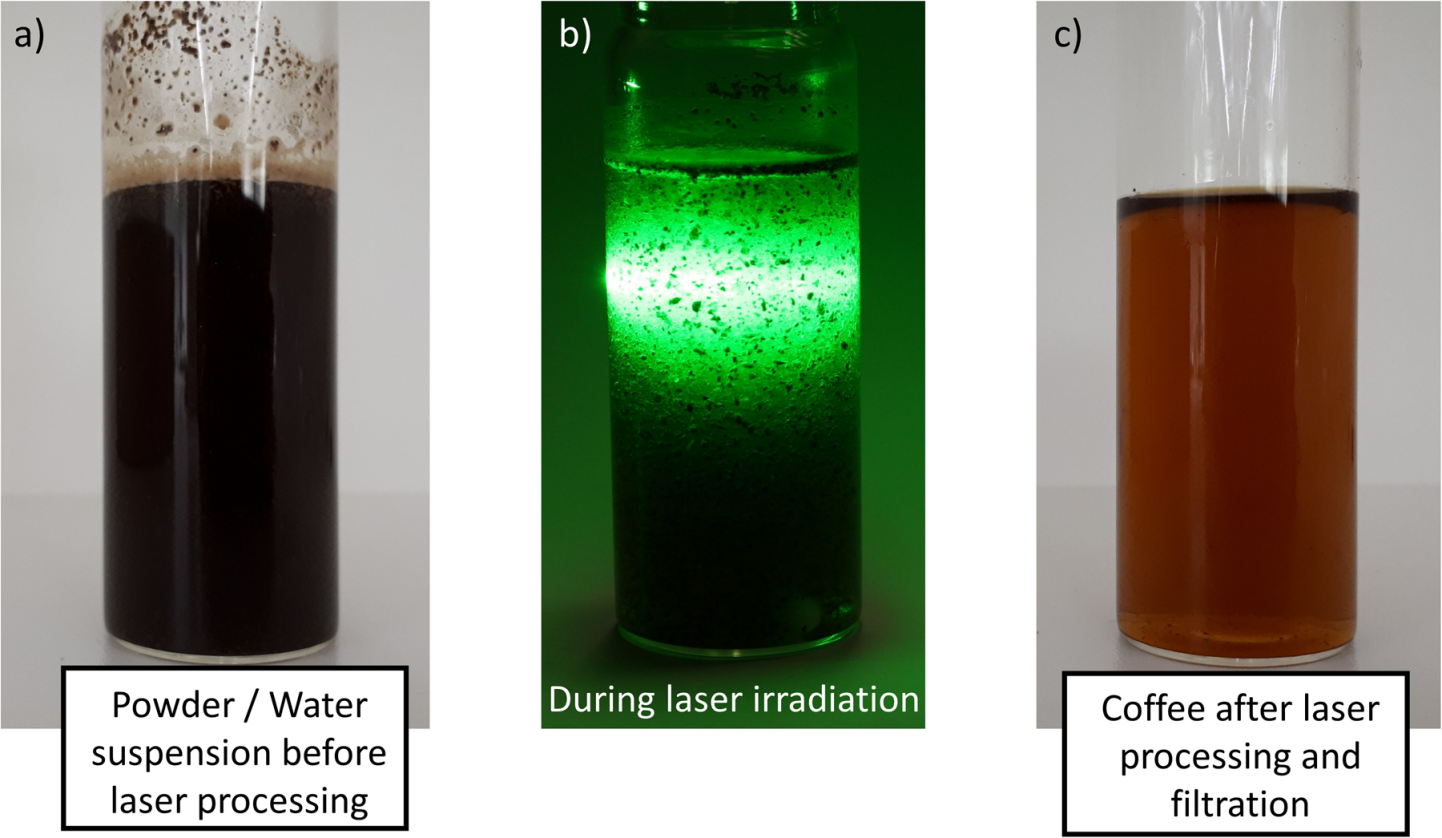
Coffee extraction process with an ultrashort-pulsed laser. **a** Coffee powder suspension in water, **b** Coffee powder suspension during laser irradiation, **c** Final product after 3 min of laser irradiation and subsequent filtration with commercially available coffee filters.

Pulsed laser fragmentation (laser extraction) of the coffee powder in pure water leads to a brownish-colored suspension, strongly reminiscent of a freshly brewed coffee even after filtration. Figure 1a–c shows the coffee along the laser irradiation process chain. A 3 min irradiation with a ps-laser leads to a temperature increase of only 5 ± 3 °C. Therefore, the temperature change during ps-LPP is neglectable, and the thermal conditions are close to the cold-brew process. Note that longer pulse durations lead to an increase in the suspension’s temperature (nanosecond pulses, data not shown). Thereby, structural changes of ingredients, such as degradation, are expected, making such pulse durations unsuitable for the cold-brewing process presented here.

### Acidity of the produced coffee

**Fig. 2:**
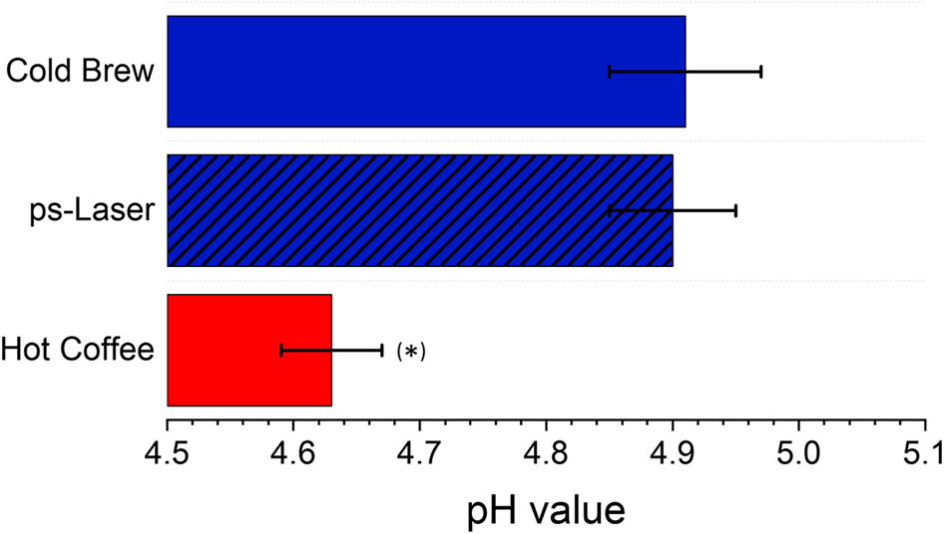
pH value (measured at 21.5 °C) of coffees after brewing, using different brewing methods. The error bars indicate the standard deviation of 13 replicates. The hot coffee’s pH value shows a significant deviation from the other coffee variants, proven by an ANOVA test.

Since lower acidity is an essential feature of cold-brewed coffee, the LPP brewing process is evaluated regarding its effect on the coffee’s pH value (Fig. 2). Note that a lower acidity of cold-brew coffee is associated with positive effects, such as reduction of gastrointestinal symptoms^1^. However, the presence of a more basic pH value in the cold-brewed variant is still under discussion^1,28^.

Since the pH value differences are relatively small, we performed a single-fraction ANOVA test. This test showed no statistical difference between the cold-brew method’s pH value and the laser-brew variant. However, if hot coffee is included in the analysis, significant differences can be observed ($F\left( {3,39} \right) = 4.813,p \le 0.03$). Therefore, we can conclude that hot-brewed coffee is slightly more acidic than cold-brewed coffee, which is in good agreement with ref. ^28^. The acidity of the ps-LPP variant is comparable to cold-brew coffee.

Besides the degree of acidity, cold-brew coffee differs from its hotter counterpart, especially in its taste^29^. Cold-brew coffee is often presented as less bitter. However, bitter is one of the most complicated tastes, and, along with sour, sweet, and salty, it is the least well understood^29^. Studies by Beauchamp et al.^30^ and further scientists^31–33^ suggest that bitter tastes can be softened in the less acidic and/or salty range as cations (e.g., Na^+^) can block the perception of bitterness. A comparable study on acidic or basic substances is currently not available. However, Sakurai et al.^34^ correlated the bitter taste of salicin with the pH of the solution and found an inhibition effect of acidic dipeptides. Even though the differences in pH value (seen in Fig. 2) are small, an influence on the taste cannot be ruled out. Note that we do not want to elucidate fundamental differences between hot and cold-brew coffee. However, the occurrence of disagreements is relevant to classify the laser-brewed variant accordingly, which requires at least the consideration of contained alkaloids (caffeine and trigonelline) and (semi-) volatile substances.

### Determination of the caffeine content

One of the best-known coffee ingredients is the alkaloid caffeine, which is described to have a bitter taste^35^. Note that caffeine is desired in coffee. It is known to cause adenosine, a neurotransmitter that promotes sleep, to be blocked while its taste is only secondarily considered. The ratio of coffee powder to water employed in practice is usually higher in cold-brew coffee than in the hot-brewed variety (accordingly, we also used the double coffee powder mass concentration, see Table 2). Hence, the higher caffeine content is expected despite the lower production temperature^7^. Please note that we aim to compare relative compositions of the different coffee variants rather than the specific concentrations. Since the temperature during ps-laser production remains close to room temperature, we have adapted the water/powder ratio to the cold-brew variant. This enables a comparison of the ps-variant with the cold-brew variant in absolute terms, but only a relative comparison to the hot coffee ingredients.

**Fig. 3:**
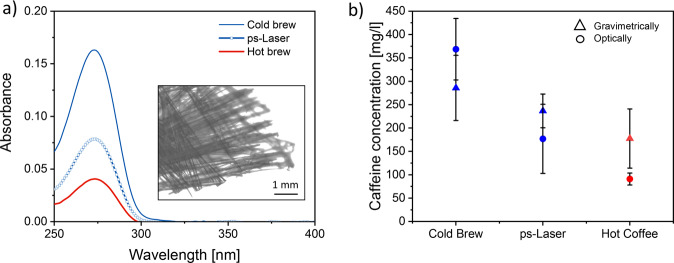
Caffeine release measured optically via UV-Vis spectroscopy. **a** UV-Vis spectra of differently brewed coffees after filtration, extraction with chloroform, evaporation of the organic phase, and dispersion of the residue in pure water. Inset: Microscopic picture of the caffeine crystals with a 10-time magnification. **b** Caffeine concentration was determined optically from the peak intensity at 273 nm and gravimetrical after extraction of the filtered coffee with chloroform, including its evaporation. The error bars indicate the standard deviation of 3 replicates.

In this study, we determine the absolute caffeine content optically and gravimetrically. In all cases, the caffeine must first be extracted from the aqueous phase (see experimental section). Since the correlation between caffeine concentration and absorbance at 273 nm shows a linear behavior, the former can be calculated based on optical measurements (Fig. 3a). The highest caffeine concentration can be found in the cold brew (Fig. 3b). Note that weighting the caffeine crystals transferred to the organic phase after extraction (Fig. 3a, inset) leads to the same result. The absolute caffeine content extracted from the powder with ps-laser pulses is somewhat lower than for the cold-brew variant and is about the same for hot coffee. However, a truly fair comparison of the different variants is only possible if we normalize various influence factors.

**Fig. 4:**
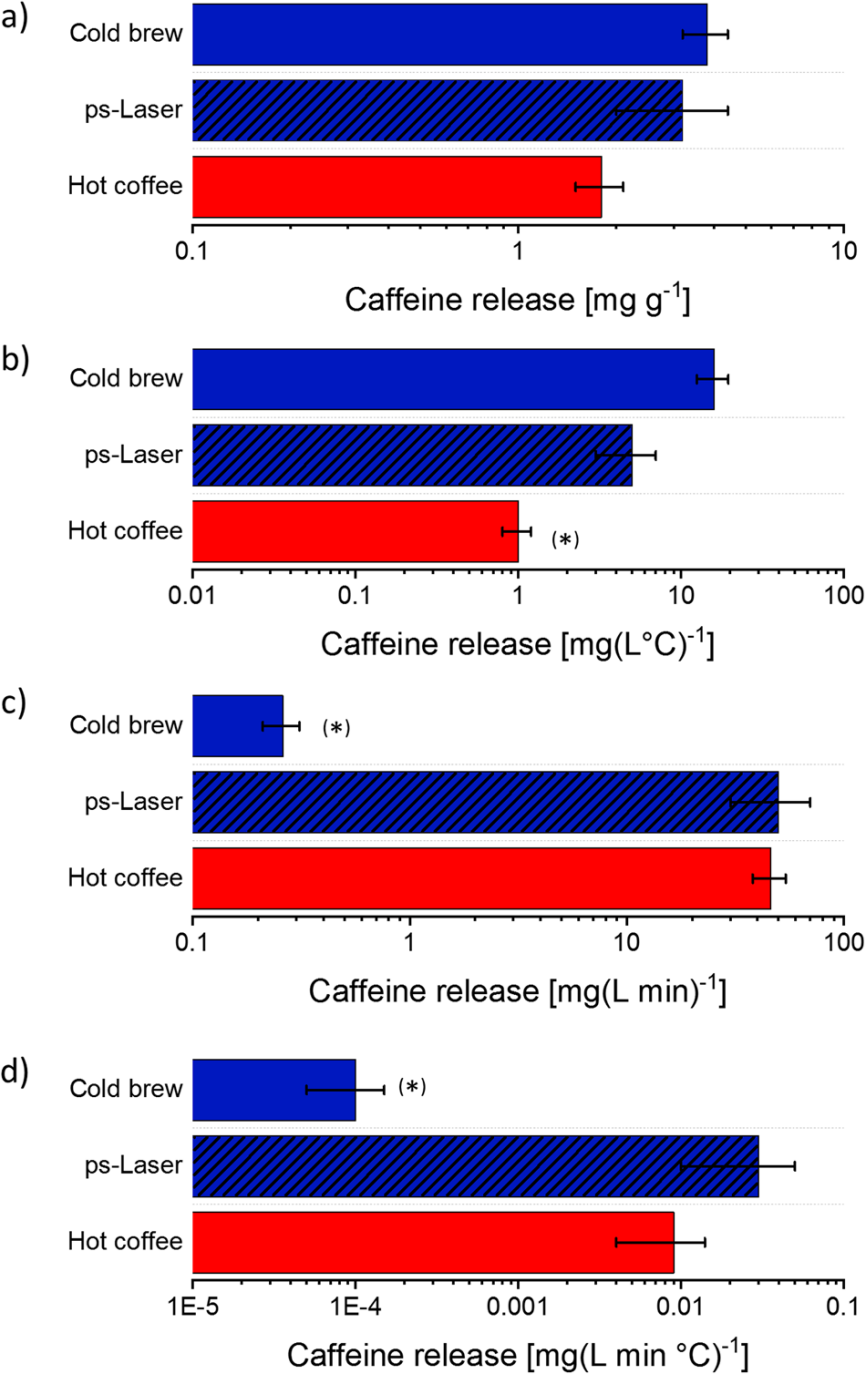
Comparison of the caffeine release efficiencies achieved by cold, hot, and laser extraction. Caffeine release calculated from the optically determined caffeine concentration and normalized to **a** coffee powder concentration, **b** temperature, **c** process duration, and **d** normalization to all parameters. The error bars indicate the standard deviation of 3 replicates. The asterisks indicate a significant deviation of this dataset from the other, calculated via an ANOVA testing.

Note that the irradiation time of 3 min can be varied. In addition, to compare the different coffees, the relative caffeine concentrations must be considered. Therefore, the optically determined caffeine concentration is normalized to the coffee powder concentration, the brewing time, and the water temperature during the process (Fig. 4). For example, the coffee powder concentration was almost twice as high as for the other brewing methods for cold brew. No significant difference between all variants can be observed after normalization to the coffee powder concentration (Fig. 4a). Considering the processing temperature leads to a clear differentiation between all three coffee variants, whereas the caffeine extraction for the cold-brew variant is far increased (Fig. 4b). However, the most interesting normalization factor, the brewing time, is not considered until Fig. 4c. Here, the inefficiency of the cold brew becomes obvious. Since it took one day to produce the cold brew, the caffeine release per min is two orders of magnitude smaller than for the other brewing methods. In Fig. 4d, it is shown that the highest relative release can be found for the ps-LPP variant, which is 3–5 times higher than for cold brew and comparable to the hot-brewed coffee. With this, cold brew cannot be considered an efficient way of coffee brewing from the viewpoint of caffeine extraction, as it takes much more time than the other methods, and much more coffee powder is needed. As mentioned above, caffeine can be considered a bitter alkaloid. Since its concentration (Fig. 4b) is higher in the two cold-produced variants, it could now be argued that these have a more bitter taste. Note that, in general, the opposite is claimed for the cold-brewed variant.

As already mentioned, bitter is the most complicated taste. Besides bitter receptor antagonists, further bitter compounds can lead to less bitterness, which correlates directly with the low amount of bitter receptors that are part of the human taste (coffee blocks 2^36^–5^35^ of the human’s 25 bitter receptors). If an antagonist or agonist occupies these receptors, the addition of other or more bitter substances can no longer lead to an increase in bitterness. So it depends on the total composition, which we will look at in the following excerpts. The further chemical analysis of the extract’s compositions shall also provide a first estimation of the extraction profiles of the three methods.

### Non-target analysis by LC-MS

**Fig. 5:**
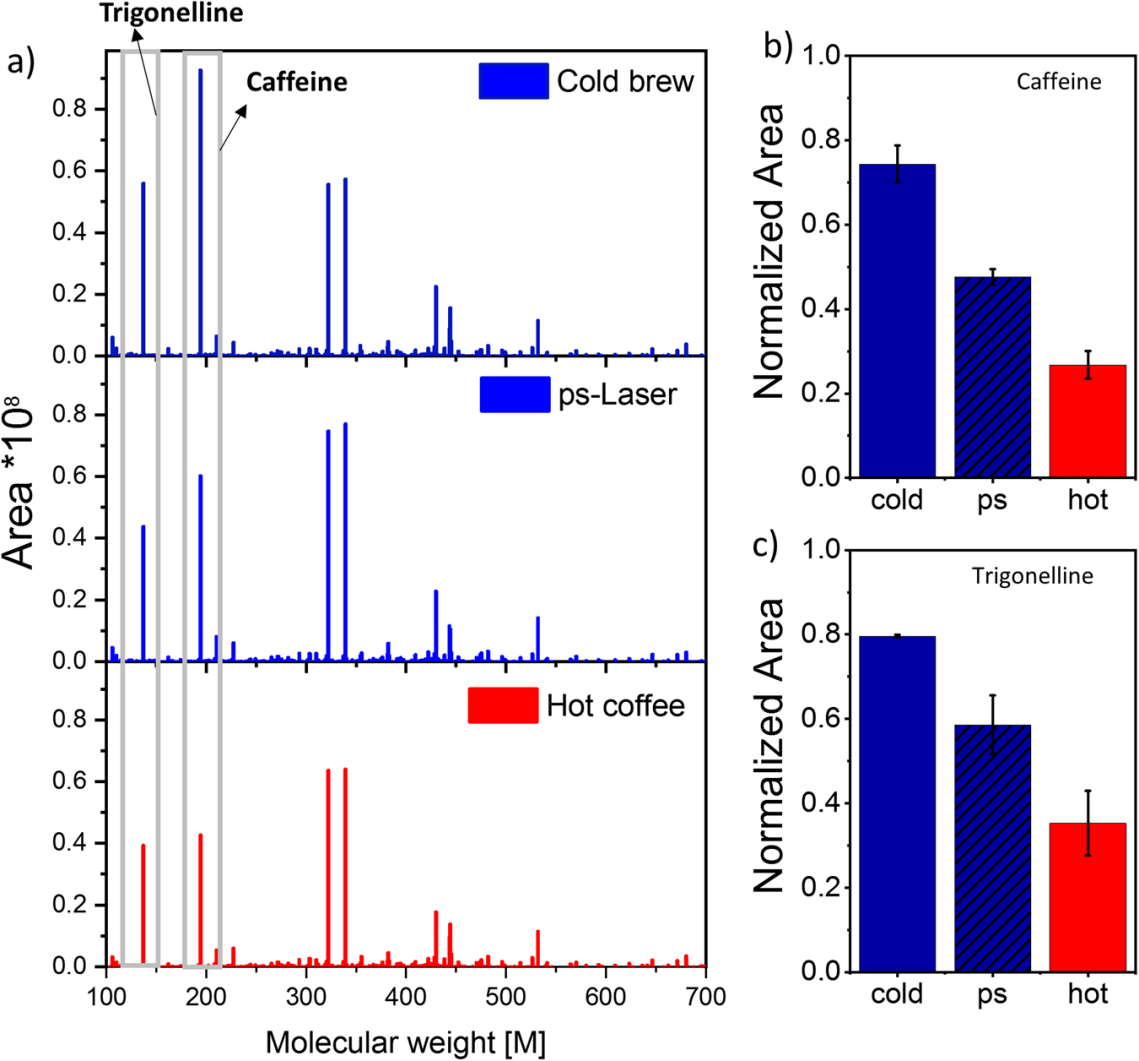
Liquid phase analysis of the as-produced coffee variants. **a** Bar plot of the LC-MS data **b** Extracted relative caffeine content, **c** Extracted trigonelline content. The error bars indicate the standard deviation of 3 replicates.

The identified features of the Liquid Chromatography Mass Spectrometry (LC-MS) analysis are given in Fig. 5a. A pure examination of those features does not reveal any differences in the general composition of all produced coffee variants in the mass range of 100–700 g/mol. Note that Fig. 5a shows the results after feature analysis.

Of course, the laser-extracted variants are particularly noteworthy here as we detected no further or even unknown substances. This points at the usefulness of ultrashort laser pulsed in LPP of organic substances, minimizing degradation compared to nanosecond laser pulses^20^. A closer look at the relative signal intensities indicates differences.

The main differences can be found between caffeine and trigonelline. Trigonelline is a well-known substance in coffee and contributes to the taste, and works further as a nutritional factor^37^. We found that the cold-brew variant has the highest trigonelline level while the hot variant shows the lowest trigonelline content. The ps-laser-extracted variant is somewhere in between; however, the detected caffeine content is also lower. As mentioned, the ps-laser method produces the coffee within 3 min (instead of 12 h). Note that quantitative analysis is hardly possible as we intended a trend analysis and performed a measurement without external calibration using authentic standards. The ratio between caffeine and trigonelline content is close for both cold brewing (classical cold brew and laser-brew) methods (amounts in both cases 1: 0.9). The lower portions in the hot brew coffee were to be expected since the coffee powder water ratio was lower than in the cold variant (see the experimental section, Table 2). Besides caffeine and trigonelline, we found ~700 other substances in the liquid phase. On the one hand, we found the flavor ingredients like *N*-butylbenzenesulfonamide equally distributed in all coffee variants. On the other hand, we found chlorogenic acid, which may be responsible for coffee’s beneficial effects on glucose regulation^38^. This indeed is more pronounced in the cold variants. Additionally, we found kahweol in all coffee variants, with an increased amount only in the cold-brew variant. The occurrence of kahweol in coffee is known^39^ and currently suspected to cause cardiovascular disease, but also an anticancer activity^39^. However, it is essential to note that we did not find any substance in the laser-generated coffee variant that did not also occur in the other coffee variants. Differences were only found in the concentration. However, since we carried out a standard-free, non-targeted measurement, it is impossible to give information on the exact concentrations.

In summary, we recorded a similar profile for all coffee variants. The cold brew and the ps-laser-brew show the highest alkaloids’ concentration, while their amount in the hot variants is decreased. Although we cannot rule out the influence of dilution for the hot brew variant, we believe the reduced amount can be referred to as thermal degradation. In the next step, the number of (semi-)volatile substances is investigated to supplement the analysis above.

### Elucidation of (semi-) volatile ingredients

In contrast to cold brew, hot-brewed coffee is known for its characteristic smell already during preparation. This olfactory aroma is almost absent when the coffee is prepared as a conventional cold brew or with a ps-laser. Although this smell is pleasant for most humans and is also part of the coffee culture, it is naturally missing in the liquid phase. This initially subjective perception already indicates that both the cold-brew coffee and the ps-laser coffee contain volatile or semivolatile components that are not evaporated during the brewing process. A selection of the identified substances is summarized in Table 1. Noticeable is pyridine, as it is only observable in the variants that are brewed near room temperature. Pyridine is known as a flavoring agent and leads to a burnt/smoky flavor^40^. Pyridine is already formed in the roasting process, during which a large proportion of trigonelline is converted to this semivolatile substance^41^. Please note that we were only able to identify pyridine in the cold variants (cold brew and laser-brew) and could not detect it in the hot-brewed coffee.

**Table 1:** Summary of substances that were detected within the head-space analysis of the produced coffee variants.

Substance	Contained in	Effect of the substance in coffee	Reference
2-Methylfuran	H/C	Health aspect	^45–47^
2-Methylbutanal	H/C	Odor, aroma	^48,49^
2,3-Pentanedione	H/C	Health aspect, flavor	^50–52^
I-Methylpyrrole	H/C	Antioxidant activity	^53,54^
Pyridine	C	Odor	^55,56^
Furan-2-carboxaldehyde	H/C	Color, flavor	^52,57–59^
2-Furanmethanol	H/C	Odor	^60,61^
2,3-butanedione	H/C	Odor	^62,63^
Thiophene	H/C	Antioxidant activity, odor	^64–67^
3-methylphenol	H/C	Odor	^68,69^
methylpyrazine	H/C	Odor	^70,71^
Ethyl acetate	H/C	Health, odor	^52,72,73^
Hexanal	H/C (without ps)	Odor via secondary products	^74–76^
2-methyl-2-Butenal	H/C	Aroma	^57,77^
2,3-Hexanedione	H/C	Health	^51^
2-(2-propenyl)- Furan	H/C	Aroma	^78^
methyl-3-Buten-1-ol	H/C	Aroma	^79^
Pyrrole	(H/C without ps)	Antioxidant activity	^53,80^
2-Pentanone	H	Aroma	^81,82^
4-Nonenoic acid	H	Aroma	^83^
2,4-dimethyl- pentanal	H	Aroma	^84^
Methyl-isobutyl-ketone	H	Aroma	^85,86^

Listed are all those substances that were measured in all repetitions of the sample but were not found in the blank. In addition, the effect of the substances in coffee is listed incl. corresponding references.

The reason that we were unable to detect pyridine in the hot coffee sample could be due to the fact that it already evaporates during the brewing process. However, pyridine starts to evaporate at >115 °C, which is 25 °C above the applied temperature in the hot variant. Another explanation for this result is based on the pH values shown in Fig. 1. The pKa of pyridine is in the range of ~5.2^42,43^ and is therefore above the measured pH value of all variants. With this, we cannot exclude that pyridine is present as cation and thereby solvated in the liquid and was not detected there due to the low molecular weight. As the pH value of the hot variant is slightly more acidic, this could lead to the more pronounced solvation of the pyridine and the associated lower detection. However, this is only an assumption and could not be verified within the experimental study. Please note that we primarily aim to study the cold variants’ comparability rather than give a perceivable sensory differentiation between hot and cold-brew coffee. However, we hope to give the impetus for the latter and look forward to further research in this field.

We can thus summarize that the chemical composition of ps-laser-extracted coffee is very similar to conventional cold-brew coffee. We have not found any significant differences in the head-space and liquid phases. Interestingly, the amount of bitter alkaloids (measured in the liquid phase) is increased in both the cold-brew and ps-LPP variant, which should intuitively lead to a more bitter taste. The taste profile that is published in many blog articles, however, corresponds exactly to the opposite. However, it should be noted that (i) we have detected a slightly more basic pH value in the cold-brewed variants, which can already have a significant influence on the taste profile, and (ii) antagonists may screen the bitter taste. Overall, the chemical profiles of the laser-extracted and conventionally cold-brew extracted coffees are quite similar, although the laser method being much faster.

## Conclusion

Cold brewing is a booming trend in coffee preparation, but patience is required as the extraction process often takes more than a working day. This may not only cause unwanted delays (or correct timing efforts) in consumption but also slows down recipe development. The use of ultrashort-pulsed lasers to produce cold-brew coffee is an elegant way to significantly shorten the cold-brewing process. The laser-brewing method reduced the production time from typically 12 h to 3 min. The use of picosecond laser fragmentation leads to a caffeine concentration of up to ~30 mg of caffeine in 100 ml of water on average, easily competing with traditional hot and cold-brewed coffee. An interesting aspect here is the normalization of the caffeine content to extraction time. Due to the very short brewing time of only 3 min, the laser-based caffeine extraction is extremely effective. Normalization to the time reveals a 300 times more effective caffeine extraction for the laser method compared to the cold-brew variant and an about three times increased caffeine extraction compared to the hot-brewed variant.

Furthermore, the identification of alkaloids revealed that the composition of ps-laser-brewed coffee is similar to cold-brewed coffee. In addition to caffeine and trigonelline, we were able to determine equal proportions of semivolatile substances. Developing this method towards a consumer product would in particular, require more in-depth and chemical analysis, to comply with food regulations, and also proband-based, statistical olfactory and tasting assessments.

We believe that our work potentially gives impetus to create a faster cold-brew enjoyment. For further improvements of the process, the laser parameters could be adjusted, and a liquid jet reactor^29^ could be used for laser post-processing upscaling minimizing powder scattering effects, which would at the same time allow continuous coffee processing, towards an automated laser-extraction coffee machine.

## Methods

**Fig. 6:**
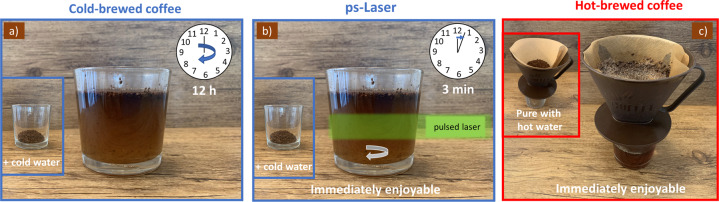
Illustration of the different brewing methods used in this work. In **a** Cold-brewed coffee and **b** ultrafast laser-extracted (ps-LPP) coffee, the ground coffee powder is mixed with cold water. While the cold-brew coffee remains unstirred for 12 h, the ps-LPP-brewed coffee is stirred and irradiated for 3 min with the pulsed laser. After 12 h (**a**) or 3 min (**b**), the coffee is filtered to remove the powder. **c** Hot brewing process. Here, the powder is placed in a filter and 90 °C hot water is poured over it and the permeate kept for analysis.

Commercially roasted and ground coffee beans (Coffee Pirates, Costa Rica SHB EP Tarrazú, 100% Arabica) were used as a starting material. The time between roasting and experiments was 10 days. The pulsed laser irradiation of 1.65 g coffee powder dispersed in 30 mL ultrapure water was performed with an Nd-YAG-laser centered at 532 nm. A ps-laser (Edgewave) with a pulse duration of 10 ps, a repetition rate of 80 kHz, and pulse energy of 125 µJ was used in the experiments. The fragmentation experiments were carried out in a small vessel equipped with a stirring system. The schematic procedure is shown in Fig. 6.

To remove excess coffee powder from the samples after laser fragmentation, commercially available coffee filters were used (Kaufland TIP, coffee filters, size 4). Please note that some filter ingredients can be flushed into the final coffee product. We included a filter step in all variants productions to keep good comparability between the different coffee variants.

As a reference for hot coffee brewing, 10 g of coffee was filled in a coffee filter. 100 ml of hot water (90 °C^26^) were slowly poured over the powder. Cold-brew coffee was made from 10 g of coffee powder and 181 ml of water, stored at room temperature for 24 h before filtration.

A summary of all process-related parameters can be found in Table 2. Please note that we prepared three replicates of each sample which makes it possible to calculate the standard deviation in different measurements in the following. Immediately after filtration, we measured the pH value and determined the caffeine concentration optically. Therefore, the caffeine was extracted from the aqueous phase by adding 10 mL chloroform (Thermo Fisher Scientific), a standard and efficient method to dissolve caffeine^21^. Impurities, which have also accumulated in the organic phase, were removed by re-extraction with a 2 M KOH (Bernd Kraft) two times. After evaporation of the chloroform, the content of caffeine was determined gravimetrically. Additionally, the caffeine was dissolved in pure water (milli-pore), and an absorbance spectrum was recorded (UV-Vis spectroscopy—Thermo Scientific, Evolution 201). By pre-calibration of pure caffeine (peak position 273 nm^44^), caffeine concentration in the respective samples can be calculated. For calibration, caffeine tablets (Coffeinum N, Mylan dura GmbH) were milled and dissolved in pure water. We performed a centrifugation step (2415 g, 2 min) to remove superfluous components and used only the supernatant for an optical caffeine calibration.

**Table 2:** Summary of the coffee brew parameters for the different coffee variants.

Short name	Coffee powder/ water [g/mL]	Coffee suspension mass concentration	Max. temperature [°C]	Contact time powder/water [min]	Other relevant process parameters
Hot brew	10/100 = 0.1	10%	90	3	
Cold coffee	10/181 = 0.055	5.5%	21.0	1440	
ps-Laser	1.65/30 = 0.055	5.5%	26.0 ± 3.0	3	10 ps, 80 kHz, 125 W, 532 nm

### Non-target LC-MS analysis

Methanol LC-MS grade was obtained from VWR International (Darmstadt, Germany). Formic acid and ammonium formate LC-MS grade were purchased from Sigma-Aldrich (St. Louis, USA). Ultrapure and deionized water with a resistivity 18.2 MΩ ∙ cm was prepared by a Sartorius Stedim water purification system (Goettingen, Germany). The coffee samples were analyzed by reversed-phase liquid chromatography coupled to high-resolution mass spectrometry. 5 µL were injected in Agilent 1260 Infinity II LC system (Agilent Technologies, Waldbronn, Germany) coupled to a Q Exactive Plus (with enhanced resolution) Orbitrap MS-System (Thermo Fisher Scientific, Bremen, Germany). Separation was conducted using a Zorbax Bonus RP RRHD (50 × 2.1 mm, 1.8 µm particles) column (Agilent Technologies, Waldbronn, Germany) at 30 °C and a binary gradient consisting of A: 5% methanol/water v/v, and B: methanol, both acidified with 0.1% formic acid and 5 mM ammonium formate. The gradient started at 0% B, linear to 95% B in 10 min and hold at 95% B for 2 min. Prior to the next injection the column was equilibrated at initial conditions for 3 min. The total run time was 15 min at a flow rate of 0.4 mL/min. Mass spectrometric detection was carried out in positive and negative ionization mode separately using the following conditions for the Heated Electrospray Ionization (HESI) source: sheath gas was 37 au, auxiliary gas was 15 au, and sweep gas was 2 au. Probe heater and capillary temperatures were set to 350 °C and 320 °C, respectively. Spray voltage was set to 3500 V in both polarities and S-Lens RF level was set to 65% in positive and 80% in negative mode. Data were acquired in a data-dependent MS/MS mode, which is fragmenting the ten most intense ions. The resolution of the full scan was 70,000 and of the MS/MS scan 17,500. Mass range was 100–1000 *m/z* with a maximum injection time of 100 ms.

Non-target data analysis was performed using the Compound Discoverer (Thermo Fisher Scientific, Waltham, USA). For feature analysis the following parameters were used: mass tolerance 5 ppm, s/n threshold 3, min peak intensity 5 × 10^5^, max retention time shift 2 min. For positive ions the following adducts were considered [M + H]^+^, [M + Na]^+^, [M + NH_4_]^+^, [M + H + NH_4_]^2+^, and for negative ions: [M + H]^−^, [M − H − H_2_O]^−^, [M − 2H]^2−^. For compound grouping, a mass tolerance of 5 ppm was applied and a retention time tolerance of less than 0.2 min. The preferred ions for fragmentation data were [M + H]^+^ and [M − H]^−^. After detection and grouping of the substances, database matching of the obtained masses, MS/MS spectra, and predicted composition was performed via mzCloud and Chemspider (Aurora Feinchemie, NIST, and NIST Spectra databases were chosen for matching). Finally, the obtained data were cleaned using mzLogic (min score 30).

### Analysis of the volatile and semivolatile components

Analysis of the (semi-) volatile components was done by in-tube extraction dynamic head-space (ITEX-DHS) followed by gas chromatography coupled to mass spectrometry (GC-MS). Sample preparation was performed on an RTC PAL autosampler (CTC Analytics, Zwingen, Switzerland) by incubating the sample at 50 °C for 15 min, shaking at 1500 rpm and subsequent ITEX-DHS extraction (ITEX Type: SilicoNert 2000 Tenax TA 80/100 mesh G23, CTC Analytics, Zwingen, Switzerland) with 55 × 1000 µL extraction strokes from the head-space at 100 µL/s. In the last extraction stroke, 1000 µL of head-space was drawn up and the components were desorbed by introducing the ITEX syringe (300 °C) in the GC injector (250 °C) and injecting 500 µL with 100 µL/s. Cleaning of the ITEX syringe was conducted at 340 °C for 13 min before and 15 min after extraction. Chromatographic separation and detection of the extracted components were implemented with a Shimadzu GCMS-TQ-2010 (Shimadzu Deutschland GmbH, Duisburg, Germany) in full scan mode (40–400 *m/z*, 0.05 s scan event time) equipped with a ZB-FFAP capillary column (50 m × 0.32 mm × 0.5 µm, Phenomenex, Torrance, USA) using helium (5.0, AirLiquide, Oberhausen, Germany) as carrier gas. The GC oven temperature started at 35 °C for 5 min, raised with 5 °C/min to 110 °C and held for 2 min, was then raised with 10 °C/min to 240 °C and held for 6 min, and finally raised with 10 °C/min to 250 °C and held for 8 min. Injector, transfer line, and electron impact ion source had a temperature of 250 °C and injection mode was splitless with a sampling and solvent cut time of 5 min. Identification of the components was done by comparing the obtained mass spectra to standard 70 eV electron impact spectra from the NIST database.

## Data Availability

The data that support the findings of this study are available from the corresponding author upon reasonable request.
